# Nonlinear magnetic vortex dynamics in a circular nanodot excited by spin-polarized current

**DOI:** 10.1186/1556-276X-9-386

**Published:** 2014-08-08

**Authors:** Konstantin Y Guslienko, Oksana V Sukhostavets, Dmitry V Berkov

**Affiliations:** 1 Depto. Física de Materiales, Facultad de Química, Universidad del País Vasco, UPV/EHU, San Sebastián 20018, Spain; 2 IKERBASQUE, Basque Foundation for Science, Bilbao 48011, Spain; 3 General Numerics Research Laboratory, Jena 07745, Germany

**Keywords:** Magnetic nanodot, Nano-oscillator, Vortex, Spin torque transfer

## Abstract

We investigate analytically and numerically nonlinear vortex spin torque oscillator dynamics in a circular magnetic nanodot induced by a spin-polarized current perpendicular to the dot plane. We use a generalized nonlinear Thiele equation including spin-torque term by Slonczewski for describing the nanosize vortex core transient and steady orbit motions and analyze nonlinear contributions to all forces in this equation. Blue shift of the nano-oscillator frequency increasing the current is explained by a combination of the exchange, magnetostatic, and Zeeman energy contributions to the frequency nonlinear coefficient. Applicability and limitations of the standard nonlinear nano-oscillator model are discussed.

## Background

Spin torque microwave nano-oscillators (STNO) are intensively studied nowadays. STNO is a nanosize device consisting of several layers of ferromagnetic materials separated by nonmagnetic layers. A dc current passes through one ferromagnetic layer (reference layer) and thus being polarized. Then, it enters to an active magnetic layer (so-called free layer) and interacts with the magnetization causing its high-frequency oscillations due to the spin angular momentum transfer. These oscillation frequencies can be tuned by changing the applied dc current and external magnetic field [1-3] that makes STNO being promising candidates for spin transfer magnetic random access memory and frequency-tunable nanoscale microwave generators with extremely narrow linewidth [4]. The magnetization in the free layer can form a vortex configuration that possesses a periodical circular motion driven by spin transfer torque [1,5-11]. For practical applications of such nanoscale devices, some challenges have to be overcome, e.g., enhancing the STNO output power. So, from a fundamental point of view as well as for practical applications, the physics of STNO magnetization dynamics has to be well understood.

**Figure 1:**
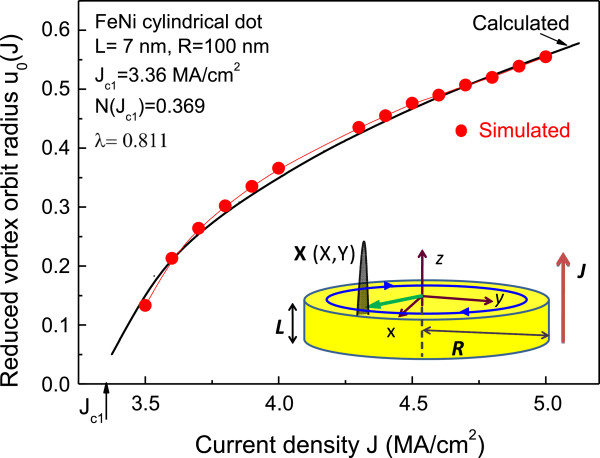
**Magnetic vortex dynamics in a thin circular FeNi nanodot.** Vortex core steady-state orbit radius *u*_0_(*J*) in the circular FeNi nanodot of thickness *L* = 7 nm and radius *R* = 100 nm vs. current *J* perpendicular to the dot plane. Solid black lines are calculations by Equation 7; red circles mark the simulated points. Inset: sketch of the cylindrical vortex state dot with the core position **X** and used system of coordinates.

In the present paper, we focus on the magnetic vortex dynamics in a thin circular nanodot representing a free layer of nanopillar (see inset of Figure 1). Circular nanodots made of soft magnetic material have a vortex state of magnetization as the ground state for certain dot radii *R* and thickness *L*. The vortex state is characterized by in-plane curling magnetization and a nanosize vortex core with out-of-plane magnetization. Since the vortex state of magnetization was discovered as the ground state of patterned magnetic dots, the dynamics of vortices have attracted considerable attention. Being displaced from its equilibrium position in the dot center, the vortex core reveals sub-GHz frequency oscillations with a narrow linewidth [2,7,12]. The oscillations of the vortex core are governed by a competition of the gyroforce, Gilbert damping force, spin transfer torque, and restoring force. The restoring force is determined by the vortex confinement in a nanodot. Vortex core oscillations with small amplitude can be well described in the linear regime, but for increasing of the STNO output power, a large-amplitude motion has to be excited. In the regime of large-amplitude spin transfer-induced vortex gyration, it is important to take into account nonlinear contributions to all the forces acting on the moving vortex. The analytical description and micromagnetic simulations of the magnetic field and spin transfer-induced vortex dynamics in the nonlinear regime have been proposed by several groups [12-22], but the results are still contradictory. It is unclear to what extent a standard nonlinear oscillator model [13] is applicable to the vortex STNO, how to calculate the nonlinear parameters, and how the parameters depend on the nanodot sizes.

In this paper, we show that a generalized Thiele approach [23] is adequate to describe the magnetic vortex motion in the nonlinear regime and calculate the nanosize vortex core transient and steady orbit dynamics in circular nanodots excited by spin-polarized current via spin angular momentum transfer effect.

## Methods

### Analytical method

We apply the Landau-Lifshitz-Gilbert (LLG) equation of motion of the free layer magnetization $\dot{m}=-\gamma m\times H_{\mathrm{eff}}+\alpha_{G}m\times \dot{m}+\gamma \tau_{s}$, where **m** = **M**/*M*_s_, *M*_s_ is the saturation magnetization, *γ* > 0 is the gyromagnetic ratio, **H**_eff_ is the effective field, and *α*_G_ is the Gilbert damping. We use a spin angular momentum transfer torque in the form suggested by Slonczewski [24], **τ**_*s*_ = *σJ***m** × (**m** × **P**), where *σ* = *ℏη*/(2|*e*|*LM*_*s*_), *η* is the current spin polarization (*η* ≅ 0.2 for FeNi), *e* is the electron charge, **P** is direction of the reference layer magnetization, and *J* is the dc current density. The current is flowing perpendicularly to the layers of nanopillar and we assume $P=P\hat{z}$. The free layer (dot) radius is *R* and thickness is *L.*

We apply Thiele's approach [23] for the magnetic vortex motion in circular nanodot (inset of Figure 1). We assume that magnetization distribution can be characterized by a position of its center **X**(*t*) that can vary with time and, therefore, the magnetization as a function of the coordinates **r** and **X**(*t*) can be written as **m**(*r*,*t*) = **m**(**r**,**X**(*t*)). Then, we can rewrite the LLG equation as a generalized Thiele equation for **X**(*t*):

(1) $$G_{\alpha \beta }{\dot{X}}_{\beta }=-\partial_{\alpha }W+D_{\alpha \beta }{\dot{X}}_{\beta }+F_{\mathrm{ST}}^{\alpha },$$

where *W* is the total magnetic energy, *α*,*β* = *x*,*y*, and ∂_*α*_ = ∂/∂*X*_*α*_. The components of the gyrotensor $\hat{G}$, damping tensor $\hat{D}$, and the spin-torque force can be expressed as follows [16]:

(2) $$\begin{array}{l} G_{\alpha \beta }\left(X\right)=\frac{M_{s}}{\gamma }\int d^{3}r\left(\partial_{\alpha }m\times \partial_{\beta }m\right)\cdot m\\ D_{\alpha \beta }\left(X\right)=-\alpha_{G}\frac{M_{s}}{\gamma }\int d^{3}r\left(\partial_{\alpha }m\times \partial_{\beta }m\right)\\ F_{\mathrm{ST}}^{\alpha }\left(X\right)=M_{s}\mathrm{\sigma J}P\cdot \int d^{3}r\left(m\times \partial_{\alpha }m\right). \end{array}$$

We assume that the dot is thin enough and **m** does not depend on *z*-coordinate. The magnetization **m**(*x*,*y*) has the components $m_{x}+im_{y}=2w/\left(1+w\bar{w}\right)$ and $m_{z}=\left(1-w\bar{w}\right)/\left(1+w\bar{w}\right)$ expressed via a complex function $w\left(\zeta ,\bar{\zeta }\right)$[25]. Inside the vortex core, the vortex configuration is described as a topological soliton, $w\left(\zeta ,\bar{\zeta }\right)=f\left(\zeta \right)$, |*f*(*ζ*)| ≤ 1, where *f*(*ζ*) is an analytic function. Outside the vortex core region, the magnetization distribution is $w\left(\zeta ,\bar{\zeta }\right)=f\left(\zeta \right)/\left|f\left(\zeta \right)\right|$, |*f*(*ζ*)| > 1. For describing the vortex dynamics, we use two-vortex ansatz (TVA, no side surface charges induced in the course of motion) with function *f*(*ζ*) being written as $f\left(\zeta \right)=-\mathrm{iC}\left(\zeta -s\right)\left(\bar{s}\zeta -1\right)/c\left(1+{\left|s\right|}^{2}\right)$[26], where *C* is the vortex chirality, *ζ* = (*x* + *iy*)/*R*, *s* = *s*_*x*_ + *is*_*y*_, **s** = **X**/*R*, *c* = *R*_*c*_/*R*, and *R*_*c*_ is the vortex core radius.

The total micromagnetic energy $W=W_{m}^{v}+W_{m}^{s}+W_{\mathrm{ex}}+W_{Z}$ in Equation 1 including volume $W_{m}^{v}$ and surface $W_{m}^{s}$ magnetostatic energy, exchange *W*_*ex*_ energy, and Zeeman *W*_*Z*_ energy of the nanodot with a displaced magnetic vortex is a functional of magnetization distribution *W*[**m**(**r**, *t*)]. Using **m** = **m**(**r**, **X**(*t*)) and integrating over-the-dot volume and surface, the energy *W* can be expressed as a function of **X** within TVA [16]. The Zeeman energy is related to Oersted field $H_{J}=\left(0,H_{J}^{\phi },0\right)$ of the spin-polarized current, *W*_*Z*_(**X**) = - *M*_*s*_ ∫ *dV***m**(**r**, **X**) ⋅ **H**_*J*_. We introduce a time-dependent vortex orbit radius and phase by *s* = *u* exp(*i*Φ). The gyroforce in Equation 1 is determined by the gyrovector $G=G\hat{z}$, where *G* = *G*_*z*_ = *G*_*xy*_. The functions *G*(*s*) and *W*(*s*) depend only on *u* = |*s*| due to a circular symmetry of the dot. *G*(0) = 2*πpM*_*s*_*L*/*γ*, where *p* is the vortex core polarity. The damping force $\hat{D}\dot{X}$ and spin-torque force **F**_*ST*_ are functions not only on *u* = |*s*| but also on direction of **s**. Nonlinear Equation 1 can be written for the circular dot in oscillator-like form

(3) $$i\dot{s}+\omega_{G}\left(u\right)s=-d\left(u\right)\dot{s}+\mathrm{i\chi}\left(u\right)s-id_{n}\left(s\right)\dot{\bar{s}},$$

where *ω*_*G*_(*u*) = (*R*^2^*u*|*G*(*u*)|)^- 1^∂*W*(*u*)/∂*u* is the nonlinear gyrotropic frequency, *d*(*u*) = - *D*(*u*)/|*G*(*u*)| is the nonlinear diagonal damping, *D* = *D*_*xx*_ = *D*_*yy*_, *d*_*n*_(*s*) = - *D*_*xy*_(*s*)/|*G*(*u*)| is the nonlinear nondiagonal damping, and *χ*(*u*) = *a*(*u*)/|*G*(*u*)|. It is assumed here that **F**_*ST*_(*s*) = *a*(*u*)(**z** × **s**) [14], where *a* is proportional to the CPP current density *J* and *a*(0) = *πRLM*_*s*_*σJ*.

To solve Equation 3, we need to answer the following questions: (1) can we decompose the functions *W*(*s*), *G*(*s*), *D*_*αβ*_(*s*), and **F**_*ST*_(*s*) in the power series of *u* = |*s*| and keep only several low-power terms? and (2) what is the accuracy of such truncated series accounting that *u* = |*s*| can reach values of 0.5 to 0.6 for a typical vortex STNO? Some of these functions may be nonanalytical functions of *u* = |*s*|. If the answer to the first question is yes, then we should decompose *W*(*s*) up to *u*^4^, **F**_*ST*_(*s*) up to *u*^3^, and *G*(*s*), *D*_*αβ*_(*s*) up to *u*^2^-terms to get a cubical equation of the vortex motion. The series decomposition of *G*(*s*) does not contain *u*^2^-term; it contains only small *c*^2^*u*^2^-term, *G*(*u*) = *G*(0)[1 - *O*(*c*^2^*u*^2^)], although *G*(*u*) essentially decreases at large *u*, when the vortex core is close to be expelled from the dot [16]. The result of power decomposition of the total energy density $w\left(u\right)=W\left(u\right)/M_{s}^{2}V$ is

(4) $$w\left(u\right)=w\left(0\right)+\frac{1}{2}\kappa u^{2}+\frac{1}{4}\kappa^{\prime }u^{4},\;u=\left|s\right|,$$

and the coefficients are

$$\begin{array}{l} \frac{1}{2}\kappa \left(\beta ,R,J\right)=8\pi \int_{0}^{\infty }\mathrm{dt}\frac{f\left(\mathrm{\beta t}\right)}{t}I^{2}\left(t\right)-{\left(\frac{L_{e}}{R}\right)}^{2}+\frac{2\pi }{15c}\frac{\mathrm{JCR\varsigma}}{M_{s}}\;\mathrm{and}\\ \frac{1}{4}\kappa^{\prime }\left(\beta ,R,J\right)=2\pi \int_{0}^{\infty }\mathrm{dt}\frac{f\left(\mathrm{\beta t}\right)}{t}\left[I_{2}^{2}\left(t\right)-I\left(t\right)I_{1}\left(t\right)\right]+\frac{1}{2}{\left(\frac{L_{e}}{R}\right)}^{2}+\frac{\pi }{15c}\frac{\mathrm{JCR}}{M_{s}}, \end{array}$$

where $I\left(t\right)=\int_{0}^{1}\mathrm{d\rho \rho}J_{1}\left(\mathrm{t\rho}\right)$, $I_{1}\left(t\right)=\int_{0}^{1}\mathrm{d\rho}\rho^{-1}{\left(1-\rho^{2}\right)}^{2}J_{1}\left(\mathrm{t\rho}\right)$, $I_{2}\left(t\right)=\int_{0}^{1}\mathrm{d\rho}\left(1+\rho^{2}\right)J_{2}\left(\mathrm{t\rho}\right)$, *β* = *L*/*R*, $L_{e}=\sqrt{2A}/M_{s}$, and *ς* = 1 + 15(ln 2 - 1/2)*R*_*c*_/8*R*.

There is an additional contribution to *κ*/2, 2(*L*_*e*_/*R*)^2^, due to the face magnetic charges essential for the nanodots with small *R*[27]. The contribution is positive and can be accounted by calculating dependence of the equilibrium vortex core radius (*c*) on the vortex displacement. This dependence with high accuracy at *cu* < < 1 can be described by the function *c*(*u*) = *c*(0)(1 - *u*^2^)/(1 + *u*^2^). Here, *c*(0) is the equilibrium vortex core radius at *s* = 0, for instance *c*(0) = 0.12 (*R*_*c*_ = 12 nm) for the nanodot thickness *L* = 7 nm.

The nonlinear vortex gyrotropic frequency can be written accounting Equation 4 as

(5) $$\omega_{G}\left(u\right)=\omega_{0}\left[1+Nu^{2}\right],$$

where the linear gyrotropic frequency is *ω*_0_ = *γM*_*s*_*κ*(*β*, *R*, *J*)/2, and *N*(*β*, *R*) = *κ*′(*β*, *R*)/*κ*(*β*, *R*).

The frequency $\omega_{0}^{\prime }=\gamma M_{s}\kappa \left(\beta ,R,0\right)/2$ was calculated in [26] and was experimentally and numerically confirmed in many papers. The nonlinear coefficient *N*(*β,R*) depends strongly on the parameters *β* and *R*, decreasing with *β* and *R* increasing. The typical values of *N*(*β,R*) at *J* = 0 are equal to 0.3 to 1.

The last term in Equation 3 prevents its reducing to a nonlinear oscillator equation similar to the one used for the description of saturated STNO in [13]. Calculation within TVA yields the decomposition $d_{n}\left(s\right)=d_{n}^{0}+d_{n}^{1}s_{x}s_{y}$, where $d_{n}^{0}=0$, i.e., the term containing *d*_*n*_(*s*) ≈ *α*_*G*_*u*^2^ <<1 can be neglected. Then, substituting *s* = *u* exp(*i*Φ) to Equation 3, we get the system of coupled equations

(6) $$\dot{\Phi }-\omega_{G}\left(u\right)=d\left(u\right)\frac{\dot{u}}{u},\;\dot{u}=\left[\chi \left(u\right)-d\left(u\right)\dot{\Phi }\right]u.$$

Equation 3 and the system (6) are different from the system of equations of the nonlinear oscillator approach [13]. Equations 6 are reduced to the autonomous oscillator equations $\dot{u}/u=\chi \left(u\right)-d\left(u\right)\omega_{G}\left(u\right)$ and $\dot{\Phi }=\omega_{G}\left(u\right)$ only if the conditions *d*^2^ < < 1 and *dχ* < < *ω*_*G*_ are satisfied and we define the positive/negative damping parameters [13] as *Γ*_+_(*u*) = *d*(*u*)*ω*_*G*_(*u*) and *Γ*_-_(*u*) = *χ*(*u*). We note that reducing the Thiele equation (1) to a nonlinear oscillator equation [13] is possible only for *axially symmetric* nanodot, when the functions *W*(*s*), *G*(*s*), *d*(*s*) and *χ*(*s*) depend only on *u* = |*s*| and the additional conditions *d*_*n*_ < < 1, *d*^2^ < < 1, and *dχ* < < *ω*_*G*_ are satisfied. The nonlinear oscillator model [13] cannot be applied for other nanodot (free layer) shapes, i.e., elliptical, square, etc., whereas the generalized Thiele equation (1) has no such restrictions.

The system (6) at $\dot{u}=0$ yields the steady vortex oscillation solution *u*_0_(*J*) > 0 as root of the equation *χ*(*u*_0_) = *d*(*u*_0_)*ω*_*G*_(*u*_0_) for *χ*(0) > *d*(0)*ω*_0_ (*J* > *J*_*c*1_) and *u*_0_ = 0 otherwise. If we use the power decompositions *ω*_*G*_(*u*) = *ω*_0_ + *ω*_1_*u*^2^, *d*(*s*) = *d*_0_ + *d*_1_*u*^2^, and *χ*(*u*) = *χ*_0_ + *χ*_1_*u*^2^ for the nonlinear vortex frequency, damping, and spin-torque terms, respectively, and account that the linear vortex frequency contains a contribution proportional to the current density $\omega_{0}\left(J\right)=\omega_{0}^{\prime }+\omega_{e}J$, where *ω*_*e*_ = (8*π*/15)(*γR*/*c*)*ς*[12,16], then we get the vortex core steady orbit radius at *J* > *J*_*c*1_

(7) $$u_{0}\left(J\right)=\lambda \left(J\right)\sqrt{J/J_{c1}-1},\;\lambda^{2}\left(J\right)=\frac{d_{0}\omega_{0}^{\prime }}{\left[d_{1}\omega_{0}\left(J\right)+d_{0}\omega_{1}\left(J\right)-\chi_{1}\left(J\right)\right]}.$$

The model parameters are $J_{c1}=d_{0}\omega_{0}^{\prime }/\left(\gamma \sigma /2-d_{0}\omega_{e}\right)$, *d*_0_ = *α*_*G*_[5 + 4 ln(*R*/*R*_*c*_)]/8, *d*_1_ = 11*α*_*G*_/6, *χ*_0_ = *γσJ*/2. The ratio *χ*_1_/*χ*_0_ = *O*(*c*^2^*u*^2^) < < 1, therefore, the nonlinear parameter *χ*_1_ can be neglected. The statement about linearity of the ST-force agrees also with our simulations and the micromagnetic simulations performed in [12,19]. The coefficient *λ*(*J*) describes nonlinearity of the system and decreases smoothly with the current *J* increasing.

### Numerical method

We have simulated the vortex motion in a single permalloy (Fe_20_Ni_80_ alloy, Py) circular nanodot under the influence of a spin-polarized dc current flowing through it. Micromagnetic simulations of the spin-torque-induced magnetization dynamics in this system were carried out with the micromagnetic simulation package MicroMagus (General Numerics Research Lab, Jena, Germany) [28]. This package solves numerically the LLG equation of the magnetization motion using the optimized version of the adaptive (i.e., with the time step control) Runge-Kutta method. Thermal fluctuations have been neglected in our modeling, so that the simulated dynamics corresponds to *T* = 0. Material parameters for Py are as follows: exchange stiffness constant *A* = 10^-6^ erg/cm, saturation magnetization *M*_s_ = 800 G, and the damping constant used in the LLG equation *α*_*G*_ = 0.01. Permalloy dot with the radius *R* = 100 nm and thickness *L* = 5, 7, and 10 nm was discretized in-plane into 100 × 100 cells. No additional discretization was performed in the direction perpendicular to the dot plane, so that the discretization cell size was 2 × 2 × *L* nm^3^. In order to obtain the vortex core with a desired polarity (spin polarization direction of dc current and vortex core polarity should have opposite directions in order to ensure the steady-state vortex precession) and to displace the vortex core from its equilibrium position in the nanodot center, we have initially applied a short magnetic field pulse with the out-of-plane projection of 200 Oe, the in-plane projection *H*_*x*_ = 10 Oe, and the duration Δ*t* = 3 ns. Simulations were carried out for the physical time *t* = 200 to 3,000 ns depending on the applied dc current because for currents close to the threshold current *J*_c1_, the time for establishing the vortex steady-state precession regime was much larger than for higher currents (see Equation 8 below).

## Results and discussion

**Figure 2:**
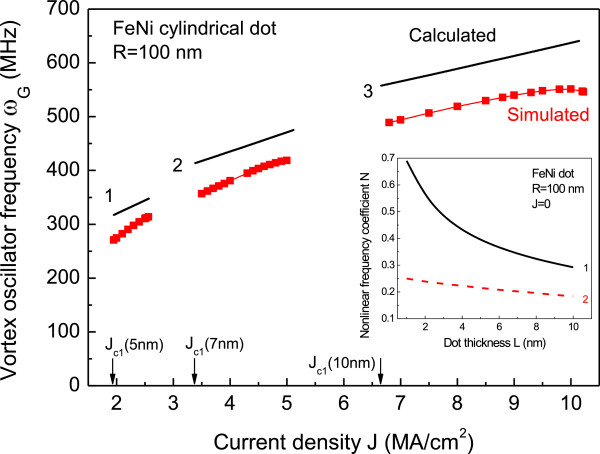
**The vortex steady-state oscillation frequency vs. current.** The nanodot thickness *L* is 5 nm (1), 7 nm (2), and 10 nm (3), and radius is *R* = 100 nm. The frequency is shown within the current range of the stable vortex steady-state orbit, *J*_*c*1_ < *J* < *J*_*c*2_. Solid black lines are calculations by Equation 5; red squares mark the simulated points. Inset: the nonlinear vortex frequency coefficient vs. the dot thickness for *R* = 100 nm and *J* = 0 accounting all energy contributions (1) and only magnetostatic contribution (2).

Calculated analytically, the vortex core steady orbit radius in circular dot *u*_0_(*J*) as a function of current *J* is compared with the simulations (see Figure 1). There is no fitting except only taking the critical current *J*_c1_ value from simulations. Agreement is quite good, confirming that all the nonlinear parameters of the vortex motion were accounted correctly. The steady orbit radius *u*_0_(*J*) allows finding the STNO generation frequency $\omega_{G}\left(J\right)=\omega_{0}\left(J\right)+\omega_{1}u_{0}^{2}\left(J\right)$, which increases approximately linearly with *J* increasing up to the second critical current value *J*_*c*2_ when the steady oscillation state becomes unstable (see Figure 2). The instability is related with the vortex core polarity reversal reaching a core critical velocity or the vortex core expelling from the dot increasing the current density *J*[12,16]. We simulated the values of *J*_*c*2_ = 2.7, 5.0, and 10.2 MA/cm^2^ for the dot thickness *L* = 5, 7, and 10 nm, respectively. The calculated STNO frequency is 15 to 20% higher than the simulated one due to overestimation of $\omega_{0}^{\prime }$ within TVA for *β* =0.1. The calculated nonlinear frequency part is very close to the simulated one, except the vicinity of *J*_*c*2_, where the analytical model fails.

Our comparison of the calculated dependences *u*_0_(*J*) and *ω*_*G*_(*J*) with simulations is principally different from the comparison conducted in a paper [19], where the authors compared Equations 5 and 7 with their simulations fitting the model-dependent nonlinear coefficients *N* and *λ* from the same simulations. One can compare Figures 1 and 2 with the results by Grimaldi et al. [20], where the authors had no success in explaining their experimental dependences *u*_0_(*J*) and *ω*_*G*_(*J*) by a reasonable model. The realistic theoretical nonlinear frequency parameter *N* for Py dots with *L* = 5 nm and *R* = 250 nm should be larger than 0.11 that the authors of [21] used. *N* = 0.25 can be calculated from pure magnetostatic energy in the limit *β* → 0 (inset of Figure 2). Accounting all the energy contributions in Equation 4 yields *N* = 0.36, which is closer to the fitted experimental value *N =* 0.50.

The system (6) can be solved analytically in nonlinear case. Its solution describing transient vortex dynamics is

(8) $$u^{2}\left(t,J\right)=\frac{u_{0}^{2}\left(J\right)}{1+\left[\frac{u_{0}^{2}\left(J\right)}{u^{2}\left(0\right)}-1\right]\exp\left(-\frac{t}{\tau_{+}\left(J\right)}\right)},$$

where *u*(0) is the initial vortex core displacement and $1/\tau_{+}\left(J\right)=2d_{0}\omega_{0}^{\prime }\left(J/J_{c1}-1\right)$ is the inverse relaxation time for *J* > *J*_*c*1_ (order of 100 ns). $u\left(t,J_{c1}\right)\propto 1/\sqrt{t}$ at t → ∞ and *J* = *J*_*c*1_. If *J* < *J*_*c*1_, the orbit radius *u*(*t*, *J*) decreases exponentially to 0 with the relaxation time $1/\tau_{-}\left(J\right)=d_{0}\omega_{0}^{\prime }\left|J/J_{c1}-1\right|$. The divergence of the relaxation times *τ*_±_ at *J* = *J*_*c*1_ allows considering a breaking symmetry second-order phase transition from the equilibrium value *u*_0_ = 0 to finite $u_{0}\left(J\right)\propto \sqrt{J/J_{c1}-1}$ defined by Equation 7. Equations 7 and 8 represent mean-field approximation to the problem and are valid not too very close to the value of *J* = *J*_*c*1_, where thermal fluctuations are important [13,21].

**Figure 3:**
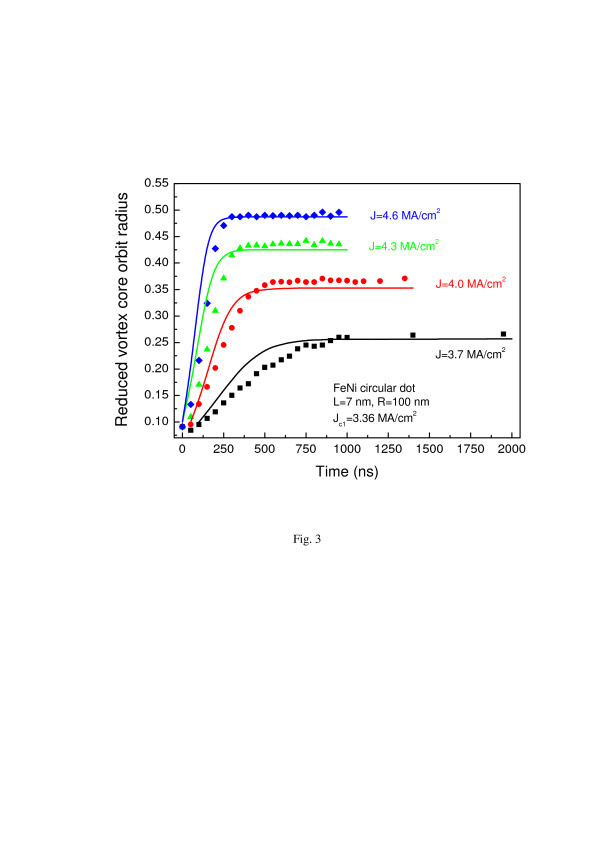
**Instant vortex core orbit radius vs. time for different currents.** The results are within the current range of the stable vortex steady-state orbit, *J*_*c*1_ < *J* < *J*_*c*2_ (5.0 MA/cm^2^). The nanodot thickness is *L* = 7 nm and the radius is *R* = 100 nm. Solid lines are calculations of the vortex transient dynamics by Equation 8, and symbols (black squares, red circles, green triangles, and blue rhombi) mark the simulated points.

Equation 8 describes approaching of the vortex orbit radius to a steady value *u*_0_(*J*) under influence of dc spin-polarized current. We note that the corresponding relaxation time is determined by only linear parameters, whereas the orbit radius (7) depends on ratio of the nonlinear and linear model parameters. The solution of Equation 8 is plotted in Figure 3 as a function of time along with micromagnetic simulations for circular Py dot with thickness *L* = 7 nm and radius *R* = 100 nm. The vortex was excited by in-plane field pulse during approximately the first 5 ns, and then the vortex core approached the stationary orbit of radius *u*_0_(*J*). We estimated *u*(0) after the pulse as *u*(0) = 0.1 and plotted the solid lines without any fitting except using the simulated value of the critical current *J*_*c*1_. Overall agreement of the calculations by Equation 8 and simulations is quite good, especially for large times *t* ≥ 3*τ*_+_, although the calculated relaxation time *τ*_+_ is smaller than the simulated one due to overestimation of $\omega_{0}^{\prime }$ within TVA. The typical simulated ratio *J*_*c*2_/*J*_*c*1_ ≈ 1.5; therefore, minimal *τ*_+_ ≈ 20 to 30 ns. But the transient time of saturation of *u*(*t*, *J*) is about of 100 ns and can reach several microseconds at *J*/*J*_*c*1_ < 1.1. The simulated value of *λ* = 0.83, whereas the analytic theory based on TVA yields the close value of *λ*(*J*_*c*1_) = 0.81.

Typical experiments on the vortex excitations in nanopillars are conducted at room temperature *T* = 300 K without initial field pulse, i.e*.*, a thermal level *u*(0) should be sufficient to start vortex core motion to a steady orbit. To find the thermal amplitude of *u*(0), we use the well-known relation between static susceptibility of the system $\hat{\chi }$ and magnetization fluctuations ${\langle M_{\alpha }^{2}\rangle }_{T}-{\langle M_{\alpha }\rangle }_{T}^{2}=\left(k_{B}T/V\right)\chi_{\alpha \alpha }$. The in-plane components are $\chi_{\mathrm{xx}}=\chi_{\mathrm{yy}}=\left(\xi^{2}/2\right)\left(\gamma M_{s}/\omega_{0}^{\prime }\right)$, and **M** = *ξM*_*s*_**s**, where *ξ* = 2/3 within TVA [26]. This leads to the simple relation ${\langle u^{2}\rangle }_{T}=\left(k_{B}T/M_{s}^{2}V\right)\left(\gamma M_{s}/\omega_{0}^{\prime }\right)$. It is reasonable to use $u_{T}\left(0\right)=\sqrt{{\langle u^{2}\rangle }_{T}}$ for interpretation of the experiments. *u*_*T*_(0) ≈ 0.05 (5 nm in absolute units) for the dot made of permalloy with *L* = 7 nm and *R* = 100 nm.

The nonlinear frequency coefficient *N*(*β*, *R*, *J*) = *κ*′(*β*, *R*, *J*)/*κ*(*β*, *R*, *J*) is positive (because of *κ*, *κ*′ >0 for typical dot parameters), and it is a strong function of the dot geometrical sizes *L* and *R* and a weak function of *J*. For the dot radii *R* > > *L*_*e*_, *N*(*β*, *R*, 0) ≈ 0.21 - 0.25 (the magnetostatic limit, see inset of Figure 2). If *R* > > *L*_*e*_ and *β* → 0, then *N*(*β*, *R*, 0) ≈ 0.25 [14]. For the realistic sizes of free layer in a nanopillar (*R* is about 100 nm and *L* = 3 to 10 nm), this coefficient is essentially larger due to finite *β* and exchange contribution, and it can be of order of 1. The exchange nonlinear contribution *κ*′_ex_ is important for *R* < 300 nm. However, the authors of [19-21] did not consider it at all. Note that *N*(0.089, 300 *nm*, 0) ≈ 0.5 recently measured [29] is two times larger than 0.25. The authors of [19] suggested to use an additional term ~*u*^6^ in the magnetic energy fitting the nonlinear frequency due to accounting a *u*^4^-contribution (*N* = 0.26) that is too small based on [14], while the nonlinear coefficient *N*(*β*, *R*) calculated by Equation 5 for the parameters of Py dots (*L* = 4.8 nm, *R* = 275 nm) [19] is equal to 0.38. Moreover, the authors of [19] did not account that, for a high value of the vortex amplitude *u* = 0.6 to 0.7, the contribution of nonlinear gyrovector *G*(*u*) ∝ *c*^2^*u*^2^ to the vortex frequency is more important than the *u*^6^-magnetic energy term. The gyrovector *G*(*u*) decreases essentially for such a large *u* resulting in the nonlinear frequency increase. The TVA calculations based on Equation 5 lead to the small nonlinear Oe energy contribution *κ*′_Oe_, whereas Dussaux et al. [19] stated that *κ*′_Oe_ is more important than the magnetostatic nonlinear contribution.

## Conclusions

We demonstrated that the generalized Thiele equation of motion (1) with the nonlinear coefficients (2) considered beyond the rigid vortex approximation can be successfully used for quantitative description of the nonlinear vortex STNO dynamics excited by spin-polarized current in a circular nanodot. We calculated the nonlinear parameters governing the vortex core large-amplitude oscillations and showed that the analytical two-vortex model can predict the parameters, which are in good agreement with the ones simulated numerically. The Thiele approach and the energy dissipation approach [12,19] are equivalent because they are grounded on the same LLG equation of magnetization motion. The limits of applicability of the nonlinear oscillator approach developed for saturated nanodots [13] to vortex STNO dynamics are established. The calculated and simulated dependences of the vortex core orbit radius *u*(*t*) and phase Φ(*t*) can be used as a starting point to consider the transient dynamics of synchronization of two coupled vortex ST nano-oscillators in laterally located circular nanopillars [30] or square nanodots with circular nanocontacts [31] calculated recently.

## Competing interests

The authors declare that they have no competing interests.

## Authors' contributions

KYG formulated the problem and carried out the analytical calculations. OVS and DVB conducted the micromagnetic simulations. KYG supervised the work and finalized the manuscript. All authors have read and approved the final manuscript.
